# Supplementation of sperm cooling medium with Eurycoma longifolia extract enhances native Thai chicken sperm quality and fertility potential

**DOI:** 10.3389/fvets.2024.1474386

**Published:** 2024-09-04

**Authors:** Thirawat Koedkanmark, Ruthaiporn Ratchamak, Supakorn Authaida, Wuttigrai Boonkum, Yoswaris Semaming, Vibuntita Chankitisakul

**Affiliations:** ^1^Department of Animal Science, Faculty of Agricultural, Khon Kaen University, Khon Kaen, Thailand; ^2^Network Center for Animal Breeding and Omics Research, Khon Kaen University, Khon Kaen, Thailand; ^3^Major of Animal Science, Department of Agricultural Technology, Faculty of Technology, Mahasarakham University, Maha Sarakham, Thailand; ^4^Program in Veterinary Technology, Faculty of Technology, Udon Thani Rajabhat University, Udon Thani, Thailand

**Keywords:** cold semen preservation, antioxidant, Long Jack, rooster semen, lipid peroxidation

## Abstract

Cooled semen storage methods result in oxidative stress generated by an imbalance between oxidation rates, specifically reactive oxygen species production, and sperm cell antioxidants, leading to degradation of semen quality. We aimed to investigate the impact of adding Eurycoma longifolia (EL) extract as an antioxidant supplement in semen storage medium (IGGKPh semen extender) on semen quality and fertility potential. EL extract at concentrations of 5, 10, 15, and 20 mg/mL was assessed for its antioxidant capacity in IGGKPh semen extender. Our findings revealed that the total phenolic content in the EL extract did not vary significantly across the various concentrations and temperatures tested. However, incubation at 5°C was found to be the most effective temperature for increasing the EL extract antioxidant capacity as assessed via the 2,2-diphenyl-1-picrylhydrazyl inhibition assay in a dose-dependent manner. Supplementation of the IGGKPh semen extender with 15 mg/mL EL extract was found to enhance semen quality during cold storage for up to 48 h (*p* < 0.05), as evidenced by decreased malondialdehyde levels in cooled semen (*p* < 0.05). However, antioxidant enzyme activities showed no significant differences among the various experimental groups (*p* > 0.05). The fertility test showed that the 15 mg/mL EL extract group stored for 24 h had a higher percentage than the control group (*p* < 0.05). However, there was no significant difference in percentage between the two groups at 48 h of storage (*p* > 0.05). The hatchability showed no significant difference in both 24 and 48-h storage periods (*p* > 0.05). Our results indicated that supplementing the IGGKPh semen extender with 15 mg/mL EL extract may positively influence semen quality during storage, suggesting potential applications for enhancing semen quality.

## 1 Introduction

Artificial insemination (AI) has become widely utilized in commercial turkey production due to their substantial body size (1), whereas its adoption in the broiler industry remains limited, primarily due to the labor-intensive breeding practices involved. However, in the context of native Thai chicken production, AI has gained popularity within battery cage systems for parent stocks. This method offers advantages such as enhanced ease of management and increased mating ratios (2). Implementation of AI in native Thai chicken has the potential to elevate the male-to-female ratio during natural mating from 1:10 to 1:25, resulting in economic benefits through reduced male requirements (3).

Semen viability is a critical concern for Thai farmers, as semen starts to lose fertility beyond 1-h post-collection due to dehydration (4). Rural farmers often utilize a saline (0.9% NaCl) solution as a short-term diluent in a 1:3 ratio to sustain sperm viability for immediate use. However, extended storage for 24 h at 25°C proves impractical with saline diluents (5). For optimal semen preservation over extended periods, dilution with suitable semen extenders, and storage at 2–5°C is necessary to minimize sperm metabolism (6). It has been reported that semen fertility decreases with semen stored for 24 h or longer (7), possibly due to oxidative stress incurred during storage (8, 9).

The oxidative stress attributed to semen storage significantly impacts sperm function, with an imbalance between antioxidants and free radicals exacerbating the decline in fertility parameters. The accumulation of malondialdehyde (MDA), an end product of lipid peroxidation, exhibits a marked increase after 24 h of semen storage at 4°C compared to MDA levels in fresh semen (10). Concurrently, antioxidant enzyme activity within semen diminishes progressively during the storage period (11). An increase in reactive oxygen species (ROS) levels in stored semen directly compromises sperm viability, acrosome integrity, mitochondrial potential, and DNA integrity, ultimately reducing semen fertility potential (12). Therefore, an antioxidant that would act as a scavenger of ROS is required to be added to the semen extender to improve sperm quality and fertility during semen storage and processing.

Plant extracts have emerged as natural sources of antioxidants and have shown promise in preserving and enhancing sperm function during semen storage. Eurycoma longifolia (EL), commonly known as ‘Tongkat Ali’ or ‘Long Jack’, is an herbal plant prevalent in Southeast Asia, and its root extract contains flavonoids, phenolic compounds, alkaloids, and antioxidants (13). EL extract shows a significant presence of superoxide dismutase (SOD) (14) that is pivotal for eliminating intracellular free radicals (15) and enhancing rooster sperm motility, viability, and structure preservation (16). Evidential studies in cattle species suggest that EL extract supplementation in cooled semen storage media can enhance sperm quality (17). However, its application in rooster semen preservation remains unexplored. As such, this study aimed to evaluate the antioxidant properties of the IGGKPh semen extender augmented with EL extract, assess the optimal concentration of EL extract for use as an antioxidant supplement in cooled semen storage, and determine its impact on semen quality. This involved measuring lipid peroxidation (as indicated by MDA levels) and antioxidant enzyme activities, ultimately assessing the potential effects on sperm fertility.

## 2 Methodology

### 2.1 Chemicals

The EL extract powder was obtained from Asian Bioplex Co. (Bangphra, Si-Racha, Chonburi, Thailand). It underwent a deionized water extraction process followed by spray drying. The powder exhibited a bulk density range of 0.20–0.50 g/mL, moisture content below 10%, and an active ingredient composition with a triterpenoid glycoside concentration exceeding 30%. Unless otherwise stated, all chemicals used in this study were acquired from Sigma-Aldrich (St. Louis, MO, USA).

### 2.2 Semen extender used

In this study, we used the IGGKPh semen extender for cooled semen storage experiments (18). Table 1 presents its chemical composition.

**Table 1:** Chemical composition of the IGGKPh semen extender.

Constituents	IGGKPh (g)
Potassium citrate monohydrate	0.70
Sodium glutamate	7.00
Sodium hydrogen phosphate	4.90
Sodium dihydrogen phosphate	1.05
Glucose	4.50
Inositol	4.50
mOsm	396
pH	6.9
Distilled water (mL)	500

### 2.3 Quantitative determination of antioxidants

The impact of various extraction procedures, solvents, and temperatures on the degradation of endogenous plant compounds has been recognized in the scientific literature (19, 20). Therefore, we evaluated the antioxidant properties of the EL extract in the IGGKPh semen extender. The EL extract, in a powdered form, was added to the IGGKPh semen extender at different concentrations (5, 10, 15, and 20 mg/mL) and stirred continuously to ensure a uniform distribution. The samples were then incubated at different temperatures (5, 25, and 60°C) for 6 h. Afterward, the samples were filtered and stored frozen at −20°C until analysis to ascertain the antioxidant contents quantitatively. Three replicates were prepared for each concentration of EL extract and temperature condition.

The total phenolic content (TPC) was measured in milligrams of gallic acid equivalent per gram (mg GAE/g) of the dry extract, while the antioxidant capacity was assessed using 2,2-diphenyl-1-picrylhydrazyl (DPPH) percent inhibition assay.

#### 2.3.1 Total phenolic content

Total phenolic content (TPC) is widely utilized as a crucial parameter for evaluating the overall antioxidant capacity of plants. TPC was quantified utilizing the Folin–Ciocalteu (F–C) assay (21), with gallic acid employed as a standard reference. Sample solutions for the assay were prepared at a concentration of 10 mg/mL. In parallel, calibration solutions of gallic acid were prepared at concentrations of 12.5, 25, 50, 100, 200, and 400 μg/mL in distilled water.

The reaction mixtures were then prepared by combining 50 μL of the sample solution with the F–C reagent in a 1:5 volume ratio. Subsequently, 100 μL of a 75% Na_2_CO_3_ solution was added to the mixtures and incubated at room temperature for 30 min. The absorbance of the reaction mixture was then measured at 765 nm utilizing a spectrophotometer (Microplate reader: TECAN, infinite 200 PRO, Switzerland). The inherent TPC value of samples was quantified by comparing the absorbance values to a blank. The TPC results were derived from a calibration curve constructed with gallic acid standards and were expressed in mg GAE/g of the dry EL extract.

#### 2.3.2 DPPH radical scavenging activity of the EL extract

The radical scavenging activity of the EL extract was ascertained using the DPPH radical as previously described (22). Briefly, 100 μL of a 0.2 mM DPPH radical solution in ethanol was added to 100 μL of various concentrations of the EL extract. After incubation in the dark for 30 min at room temperature, the absorbance was measured at 517 nm using a spectrophotometer (Microplate reader: TECAN, infinite 200 PRO, Switzerland). Ascorbic acid served as a positive control, and all measurements were performed in triplicate. The concentration of the EL extract that yields 50% inhibition (IC50) was calculated by plotting inhibition percentages against various extract concentrations. The inhibition percentages were computed with the formula: Inhibition percentage (%) = (A0 − A1)/A0 × 100, where A1 and A0 represent the absorbance of the DPPH radical solution after incubation with and without the EL extracts.

### 2.4 Animals and management

Twenty-five native Thai roosters (Pradu Hang Dum, 33 weeks of age) were kept in individual cages (48 × 45 × 45 cm) in an open-environment housing system. They were fed approximately 130 g of commercial feed (Balance 924, Betagro Company Limited, Thailand) that provided 17% protein, 3% fat, 6% fiber, and 13% moisture. Water was provided *ad libitum*. Semen was routinely collected weekly for AI.

Forty commercial laying hens (ISA-Brown breed, 34–40 weeks of age), with egg production >70%, were used for the fertility test. The hens were housed individually, fed approximately 120 g of the same commercial feed as the roosters, and provided with water *ad libitum*.

The Institutional Animal Care and Use Committee approved this study’s experimental protocol based on the Ethics of Animal Experimentation of the National Research Council of Thailand [Record No. IACUC-KKU-13/67; Reference No. 660201.2.11/153 (15)].

### 2.5 Semen sample collection

Rooster semen samples were collected twice a week from individual roosters into 1.5 mL microtubes containing 100 μL of IGGKPh semen extender using the dorso-abdominal massage method. This dilution step was necessary to maintain sperm viability; otherwise, dehydration due to water evaporation from the seminal plasma could occur (23). The sperm concentration reported in subsequent analyses refers to the concentration after dilution.

Following semen collection, the samples were kept at a temperature between 22 and 25°C and transported to the laboratory within 20 min of collection. Individual semen samples were assessed for mass movement and scored on a scale of 0–5 (0 = no sperm movement; 5 = very rapid waves and whirlwinds visible, with more than 90% of sperm showing forward movement). A drop of 5–10 μL semen was placed on a slide without a coverslip and examined under a compound microscope (10× magnification; Olympus CH30, Tokyo, Japan). Only semen samples with a mass movement score > 3.5 were pooled for further experimentation.

### 2.6 Pooled semen evaluation

After pooling, the semen samples were evaluated for sperm concentration and viability. Sperm concentration was evaluated using a hemocytometer chamber. Initially, 999 μL of 4% sodium chloride solution was added to 1 μL of the semen sample. The diluted semen sample was placed in the hemocytometer and examined under a compound microscope (40× magnification). The sperm concentration was determined to be 1 billion (10^9^) cells per mL. Sperm viability was determined using eosin-nigrosine staining. A 5 μL semen sample was mixed with 10 μL of eosin-nigrosine and smeared on the slide. After drying, 300 sperms were evaluated under a compound microscope (40× magnification). Sperm that appeared pink (stained with eosin) were regarded as dead, whereas sperm without any color (no penetration of eosin stain) were regarded as live. Only pooled samples meeting the following criteria were used in the experiments: sperm concentration ≥3 × 10^9^ sperm/mL, and sperm viability ≥90%.

In the present study, the pooled semen quality regarding sperm concentration and viability was 4.46 × 10^9^ ± 0.03 sperm/mL and 94.95 ± 0.85%, respectively.

### 2.7 Experimental design

To determine the optimal concentration of EL extract to be used for supplementation of semen extender during semen storage at 5°C for up to 72 h, the pooled semen was divided into five aliquots and diluted with the IGGKPh extender (1:3 v/v) containing various concentrations of EL extract: 5, 10, 15, and 20 mg/mL. An IGGKPh extender sample without EL extract supplementation served as the control. Semen quality, including total motility (MOT), progressive motility (PMOT), and sperm viability, as well as lipid peroxidation (measured by MDA) and antioxidant enzyme activities (catalase [CAT] and superoxide dismutase [SOD]), was evaluated at 0, 24, 48, and 72 h after storage (T0, T24, T48, and T72, respectively). The experiment was repeated five times.

Fertility potential was examined using the treatment group that demonstrated the most favorable outcomes after 24 and 48 h of semen storage compared with the control group through AI conducted in hens once per week for a continuous period of 4 weeks. Fertility and hatchability rates were recorded.

### 2.8 Cooled semen processing

After fresh semen evaluation, semen samples were pooled and diluted (1:3 v/v) with the IGGKPh semen extender. The diluted semen was then cooled down to 5°C within 1 h and stored for 72 h, with the semen tubes being flipped every 6 h.

### 2.9 Evaluation of sperm quality

#### 2.9.1 Sperm motility

MOT and PMOT were evaluated using a Computer-Assisted Sperm Analyser (CASA), HTM-IVOS Model 10.0 D (Hammilton-Thorne Bioscience, Beverly, MA, USA). This system was set up with the following parameters: frames per second, 60 Hz; minimum contrast, 25; minimum cell size, 10 μm. A sperm was defined as non-motile if the average path velocity was less than 5 μm/s, and sperm was considered progressively motile if the average path velocity was greater than 10 μm/s with a straightness index of 80. At least five fields of view were evaluated, with a minimum of 300 sperm per sample, to compute MOT and PMOT values.

#### 2.9.2 Sperm viability

Sperm viability was analyzed using fluorescent staining to distinguish live sperm from dead sperm based on cell membrane integrity. Two dyes were used: SYBR-14 and propidium iodide (L7011; Invitrogen, Thermo Fisher Scientific, Waltham, MA, USA). For the test, 300 μL of semen was mixed with 5 μL of SYBR-14, incubated at ~25°C for 10 min, followed by the addition of 5 μL of propidium iodide and further incubation for 5 min. The sperm were then fixed with 10% formaldehyde, and at least 300 sperm cells were counted using an IX71 fluorescence microscope (40× magnification; Olympus, Tokyo, Japan). Sperm with an intact plasma membrane exhibited green fluorescence from SYBR-14, while sperm with damaged plasma membrane stained red with propidium iodide.

#### 2.9.3 Lipid peroxidation

Lipid peroxidation was evaluated by determining the MDA concentration, the final product of lipid peroxidation, using the thiobarbituric acid reactive substances (TBARS) assay. This method involves a reaction between MDA and thiobarbituric acid (TBA), resulting in a pinkish-orange compound. In this study, the TBARS assay was performed on whole cells as described by (24). The procedure involves incubating semen with a sperm concentration of 250 × 10^6^ or a semen volume of 250–300 μL with 0.25 mL of ferrous sulfate (0.2 mM) and 0.25 mL of sodium ascorbate (1 mM), incubated at 37°C for 1 h. Following incubation, 1 mL of 15% trichloroacetic acid and 1 mL 0.375% TBA (Sigma, T550-0) were added. The mixture was heated to 100°C and placed at 4°C before centrifugation at 5,000×*g* at 4°C for 10 min to obtain the supernatant. This was used to measure the MDA concentration using UV–visible spectrophotometry (Analytikjena Model Specord 250 plus) at a wavelength of 532 nm. MDA concentration was calculated using a standard curve generated from a known concentration of MDA (0, 0.83, 1.66, 2.40, 3.33, 4.16, 4.99, 5.83, and 6.66 μmol/mL), with results expressed in μmol/mL.

#### 2.9.4 Antioxidant enzyme activity

The semen sample was centrifuged at a speed of 5,000×*g* at 4°C for 10 min to separate the sperm from seminal plasma. The seminal plasma was used to quantify the activity of two major antioxidant enzymes, namely SOD and CAT, as described previously (25).

##### 2.9.4.1 SOD activity

SOD activity was measured using a spectrophotometric assay based on the inhibition of cytochrome c reduction by xanthine oxidase. Briefly, an aliquot of seminal plasma (10 μL) was mixed with a reaction mixture containing (1 mM), xanthine (50 mM), and 155 μL of xanthine oxidase, diluted in a buffer containing sodium and EDTA at concentrations of 50 mM and 100 mM, respectively. The decrease in absorbance at 550 nm was monitored over time, and the SOD activity was calculated based on the rate of cytochrome c reduction. One unit of SOD activity is defined as the amount of enzyme required to inhibit the rate of cytochrome c reduction by 50%. The SOD activity was expressed in units per milliliter (U/mL).

##### 2.9.4.2 Catalase activity assay

Catalase activity was determined by measuring the rate of hydrogen peroxide (H_2_O_2_) decomposition using a spectrophotometer. A 10 μL sample of seminal plasma was mixed with a buffer solution containing 50 mM amino methane/EDTA and 250 mM Tris (hydroxymethyl) for a total volume of 90 μL. Then, 900 μL of 9.0 mM hydrogen peroxide (H_2_O_2_) was then added to initiate the reaction, which was incubated at pH 8.0 and 30°C for 8 min. The absorbance of the reaction mixture was measured every 5 s at 230 nm. The decrease in absorbance over time was used to calculate the rate of H_2_O_2_ decomposition, which was then expressed in units/mL (U/mL) to reflect catalase activity in the assessed seminal plasma sample.

#### 2.9.5 Sperm fertility

The fertility rate of semen in semen extender supplemented with EL extract was evaluated by AI in ISA-Brown hens (10 hens per treatment group) as described previously (26). Using a tuberculin syringe (1 mL), 0.1 mL of cooled semen, with a concentration of approximately 100 × 10^6^ sperm per insemination dose, was deposited into the cloaca, approximately 4 cm deep. AI was performed once per week and continued for 4 weeks. Fertility assessment was conducted by collecting eggs from Day 2 after the first insemination until Day 8 following the last insemination. Eggs were stored on paper trays at a controlled temperature (22–25°C) and incubated weekly to determine fertility status on Day 7 using candling. The fertility rate was calculated as the percentage of fertile eggs out of the total number of incubated eggs. Hatchability rate, defined as the percentage of hatched eggs from fertile.

### 2.10 Statistical analysis

To determine the optimal concentration of EL extract to be used for supplementation of semen extender during cooled semen storage at 5°C for up to 72 h, a split-plot design with five replicates was used with two factors (treatment and storage time). Five treatments were randomly arranged in the main plot, and storage time at 5°C with four storage durations (0, 24, 48, and 72 h) were randomly distributed subplots.

The fertility potential was analyzed using a group t-test analysis, encompassing two treatments: a control group and EL extract supplementation in semen extender at a dose of 15 mg/mL.

All data underwent assessment for a normal distribution using univariate analysis and the Shapiro–Wilk test. Various parameters such as total motility, progressive motility, viability, mitochondrial potential, cell apoptosis, lipid peroxidation level, antioxidant enzyme activity, fertility, and hatchability were analyzed using analysis of variance via SAS software (SAS Institute, Inc., Cary, NC, USA). The mean values for each treatment group were compared using Tukey’s test. Orthogonal polynomial contrasts were used to determine linear and quadratic responses to each treatment. Data were considered to be statistically significant at *p* < 0.05.

## 3 Results

### 3.1 Quantitative determination of antioxidants

Based on the data presented in Table 2, the TPC in semen extenders ranged from 4 to 7 mg GAE/g and did not exhibit variability based on the concentration of EL extract or the incubation temperatures. Conversely, the antioxidant capacity, as determined by DPPH inhibition, was found to be concentration-dependent and influenced by the temperature of incubation. Higher concentrations of the extract led to increased antioxidant capacity, as indicated by higher antioxidant capacity values at 20 mg/mL compared to those observed at lower concentrations of the extract at 5°C of incubation. Additionally, the temperature of incubation appeared to influence antioxidant capacity, with lower antioxidant capacity values observed at 25°C and 60°C compared with those seen at 5°C, and some values even display negative outcomes.

**Table 2:** Analysis of antioxidant properties of EL extract.

EL extract supplementation dosage in IGGKPh semen extender	Temperature of incubation (°C)		
	5	25	60
	**Total phenolic content (mg GAE/g)**		
5 mg/mL	5.87 ± 0.28	4.92 ± 0.21	4.70 ± 0.28
10 mg/mL	7.73 ± 0.74	6.05 ± 0.42	5.26 ± 0.49
15 mg/mL	6.50 ± 0.34	6.39 ± 0.46	5.00 ± 0.32
20 mg/mL	6.14 ± 0.17	5.20 ± 0.39	5.70 ± 0.24
	**Antioxidant capacity DPPH (%) inhibition**		
Ascorbic acid (control)	87.32 ± 1.26		
5 mg/mL	40.95 ± 1.59	6.25 ± 0.64	−46.03 ± 2.43
10 mg/mL	65.29 ± 0.64	19.29 ± 2.20	−20.34 ± 1.65
15 mg/mL	81.97 ± 0.49	27.88 ± 0.64	−43.32 ± 2.12
20 mg/mL	91.87 ± 0.50	−78.93 ± 0.96	−53.70 ± 2.67

DPPH, 2,2-diphenyl-1-picrylhydrazyl; GAE, gallic acid equivalent.

### 3.2 Effects of EL extract on cooled semen storage

#### 3.2.1 Sperm quality parameters

The effects of the EL extract on semen quality after storage at 5°C for 72 h are presented in Table 3. The interaction effect between treatment and storage time was found to be significant for all sperm parameters (*p* < 0.05). Most sperm quality parameters continued to decrease as storage time increased (*p* < 0.05).

**Table 3:** Effects of EL extract treatments for various storage times (at 5°C for 0, 24, 48, and 72 h) on rooster semen quality (mean ± SEM).

Duration of storage (h)	EL extract treatment dosage					SEM	*p*-value				
	Control	5 mg/mL	10 mg/mL	15 mg/mL	20 mg/mL		Treatment	Time of storage	Interaction	Linear	Quadratic
MOT (%)											
T0	87.84^aw^	87.66^aw^	88.27^aw^	88.16^aw^	85.34^bw^	1.89	<0.0001	<0.0001	0.0068	0.5484	<0.0001
T24	72.33^cx^	72.54^cx^	74.09^bx^	76.90^ax^	68.32^dx^	1.50					
T48	67.50^by^	67.62^by^	71.36^ay^	71.26^ay^	65.80^cy^	0.76					
T72	53.84^dz^	55.19^cz^	62.70^bz^	65.77^az^	50.92^ez^	0.60					
PMOT (%)											
T0	81.81^bw^	81.23^bw^	84.03^aw^	83.94^aw^	81.44^bw^	1.09	0.0003	<0.0001	0.0458	0.4870	0.0170
T24	64.45^cx^	64.32^cx^	67.03^bx^	70.19^ax^	62.19^dx^	0.90					
T48	55.21^cy^	54.71^cy^	58.71^by^	61.43^ay^	54.42^cy^	1.04					
T72	53.41^az^	53.65^az^	54.27^az^	56.40^az^	53.94^az^	3.14					
Viability (%)											
T0	81.90^bw^	81.33^bw^	85.58^aw^	86.42^aw^	81.24^bw^	2.83	<0.0001	<0.0001	<0.0001	0.0324	<0.0001
T24	74.42^bx^	76.45^bx^	81.63^ax^	83.78^ax^	75.69^bx^	2.75					
T48	73.03^cy^	73.72^cy^	76.20^by^	80.56^ay^	73.95^cy^	2.52					
T72	69.35^cz^	70.90^cz^	73.82^bz^	75.81^az^	68.68^cz^	0.85					

Values within a row with different superscript letters ^(a, b, c, d, e)^ indicate significant differences between treatments; values within a column with different superscript letters ^(w, x, y, z)^ indicate significant differences between storage times. *p* < 0.05. MOT, total motility; PMOT, progressive motility. T0 = 0 h, T24 = 24 h, T48 = 48 h, T72 = 72 h after storage.

Our results showed significant differences in semen quality between the control and the groups supplemented with EL extract. At T0, the 10 mg/mL and 15 mg/mL EL extract groups displayed significantly higher PMOT and viability compared to the other EL extract groups and the control group (*p* < 0.05).

At T24, the 15 mg/mL group exhibited significantly higher MOT and PMOT than the 10 mg/mL group (*p* < 0.05), but no significant difference was observed in viability (*p* > 0.05). However, the viability of the 15 mg/mL group was higher than that of the 10 mg/mL group at T48 and T72 h (*p* < 0.05), while PMOT differed statistically between these groups only at T48 but not at T72.

The 5 mg/mL EL extract group did not exhibit significant differences in sperm parameters compared to the control group (*p* > 0.05). In contrast, the 20 mg/mL EL extract group exhibited a detrimental effect, with lower sperm motility at all storage times (*p* < 0.05).

Furthermore, orthogonal polynomial contrast analysis revealed a significant quadratic relationship (*p* < 0.05) between sperm quality parameters and KP extract concentration within each experimental period. This indicates that sperm quality increased with increasing EL extract concentrations, with the highest sperm quality observed at 15 mg/mL.

#### 3.2.2 Lipid peroxidation and antioxidant enzyme activity

The results of lipid peroxidation, as indicated by MDA concentration, and the activities of the antioxidant enzymes SOD and CAT are presented in Table 4. At T0, MDA levels did not show significant differences among treatments (*p* > 0.05). At T24, significantly lower MDA levels were observed in the groups supplemented with 15 mg/mL and 20 mg/mL of EL extract compared to the control group (*p* < 0.05). At T48, all EL extract groups (5 mg/mL, 15 mg/mL, and 20 mg/mL) showed significantly lower MDA levels compared to the control group (*p* < 0.05). MDA decreased linearly with increasing concentration levels of EL extract (*p* < 0.05). No significant differences were noted at T72 storage time (*p* > 0.05). In contrast, the SOD and CAT levels across the different groups did not exhibit significant differences at any of the storage times (*p* > 0.05).

**Table 4:** Effects of EL extract treatments for various storage times (at 5°C for 0, 24, 48, and 72 h) on lipid peroxidation (malondialdehyde [MDA]), catalase (CAT), and superoxide dismutase (SOD) enzyme activities.

Duration of storage (h)	EL extract treatment dosage					SEM	*p*-value				
	Control	5 mg/mL	10 mg/mL	15 mg/mL	20 mg/mL		Treatment	Time of storage	Interaction	Linear	Quadratic
MDA (μmol/mL)											
T0	0.59^aw^	0.72^aw^	0.66^aw^	0.59^aw^	0.60^aw^	0.11	<0.0001	0.0096	0.4970	0.0009	0.4725
T24	1.28^ax^	1.31^ax^	0.92^bx^	0.60^cx^	0.63^cx^	0.05					
T48	2.40^ay^	1.72^by^	1.56^by^	1.55^by^	1.91^by^	0.14					
T72	2.52^az^	2.38^az^	2.30^az^	2.38^az^	2.54^az^	0.16					
SOD (U/mL)											
T0	4.07	3.91	4.07	4.11	3.96	0.08	0.3458	0.4934	0.8410	0.6963	0.1289
T24	4.03	4.00	4.14	4.09	3.98	0.08					
T48	3.97	4.08	3.97	4.02	3.90	0.11					
T72	3.96	3.96	4.25	4.11	4.13	0.08					
CAT (U/mL)											
T0	21.05	21.28	21.39	19.59	21.15	0.65	0.268	0.3189	0.7293	0.7297	0.7029
T24	20.17	20.10	20.65	20.07	21.28	0.58					
T48	21.58	21.05	22.06	19.80	21.35	0.59					
T72	19.53	21.22	20.90	19.95	21.35	0.23					

Values within a row with different superscript letters ^(a, b, c, d, e)^ indicate significant differences between treatments; values within a column with different superscript letters ^(w, x, y, z)^ indicate significant differences between storage times. *p* < 0.05. T0 = 0 h, T24 = 24 h, T48 = 48 h, T72 = 72 h after storage.

#### 3.2.3 Effects of EL extract on sperm fertility

Based on our sperm quality and lipid peroxidation results, supplementing the semen extender with 15 mg/mL of EL extract for a storage duration of 24–48 h was chosen as the treatment for further investigation into fertility.

The effects of the EL extract on fertility and hatchability values after storage at 5°C for 48 h are presented in Table 5. At a storage duration of 24 h (T24), the fertility rate in the EL extract group was higher than that of the control groups (*p* < 0.05) but was not significantly different between various treatment groups at T48 (*p* > 0.05). The hatchability values were not significantly different between groups at the same storage time (*p* > 0.05).

**Table 5:** Effects of EL extract treatment on fertility and hatchability rates after artificial insemination.

Parameters	Treatments					
	T24			T48		
	Control	15 mg/mL	*p*-value	Control	15 mg/mL	*p*-value
No. of eggs	239	218		200	196	
Fertility^1^ (%)	73.59^a^	83.10^b^	0.0440	59.80	60.15	0.9325
Hatchability^2^ (%)	73.03	73.27	0.9745	63.57	66.30	0.5675

^a,b^ Different letters within a row of the same storage period indicate significant differences. p < 0.05. T24 = 24 h, T48 = 48 h after storage.

1 Fertility rate was calculated as the percentage of fertile eggs out of the total number of incubated eggs.

2 Hatchability rate was defined as the percentage of hatched eggs from fertile.

## 4 Discussion

Cooled rooster semen storage at 2–5°C is recommended to minimize sperm metabolism without inducing serious cold shock. However, it has been reported that the amount of ROS tends to increase gradually during cold storage (27). With the passage of semen storage time, this increase in ROS can lead to plasma membrane dysfunction, ultimately resulting in a reduced fertility potential when semen is stored for extended periods, such as 24 h or longer. In response to this challenge, supplementing the semen extender medium with EL extract at a concentration of 15 mg/mL has proven beneficial as an antioxidant substance. This supplementation has demonstrated the capacity to enhance semen quality during cooled storage for up to 48 h. Interestingly, a decrease in MDA levels, an indicator of lipid peroxidation, was noted in the EL extract treatment groups, even though antioxidant enzyme activities did not show significant differences among the groups. Our data revealed that the EL extract treatment groups exhibited increased sperm fertility rates for a cooled semen storage duration of 24 h. In addition, it is important to note that higher doses of EL extract supplementation have been observed to negatively affect sperm quality despite having greater antioxidant capacity (Table 2), indicating the need to establish an optimal EL extract dosage level to achieve the desired improvements in semen quality during cooled storage.

The extraction procedures, solvents, and temperatures utilized can significantly impact the degradation of endogenous plant compounds (19, 20). In the present study, we investigated the antioxidant properties of EL extract used to supplement the semen extender. While the levels of TPC (total phenolic content) in the various EL extract concentrations employed (between 5–20 mg/mL) and incubation temperatures used (5°C, 25°C, and 60°C) did not show a significant difference, ranging from 4.7 to 7.7 mg GAE/g, our findings suggest that TPC levels may not be markedly affected by EL extract dosage and incubation temperature. This observation is likely due to the limited solubility of phenolic compounds in water. The reported solubility of TPC in water from EL is 7.3229 mg GAE/g (28), which aligns with our findings (Table 2), showing consistent TPC levels in the EL extract within the semen extender despite variations in EL extract concentrations. The EL extract used in this study was prepared using a deionized water extraction method and incorporated into a distilled water base in the semen extender, reinforcing the idea that the solubility limitation of TPC in water is inherent to EL extract usage.

While previous studies have reported a decrease in phenolic and flavonoid contents in other plant extracts at elevated temperatures (29–33), our study did not observe a significant impact on total phenolic content at higher temperatures (up to 60°C). It’s possible that elevated temperature affects certain phenolic compounds, as suggested by reports where exceeding 130°C during spinach extraction resulted in decreased flavonoid levels while the phenolic content remained unaffected (30). Similarly, extracting peach fruit at temperatures above 60°C caused a reduction in flavonoid levels while phenolic content remained stable (32). This suggests that EL extract might possess unique thermal stability (at temperatures below 60°C and incubation times less than 6 h, as used in this study) compared to other plant extracts.

However, despite this thermal stability, we found that temperature significantly affects the DPPH radical scavenging activity of EL extract. We demonstrated that incubation at 5°C was the most effective temperature for enhancing the antioxidant capacity, as indicated by increased percentages of DPPH inhibition in a dose-dependent manner. Conversely, as temperatures rose to 25°C and 60°C, the percentages of DPPH inhibition decreased markedly, with some reaching negative values, suggesting a loss of antioxidant properties and free radical scavenging activity. This discrepancy highlights the complexity of antioxidant activity and the need to consider multiple factors beyond just TPC. It’s possible that the temperature affects the reactivity of specific phenolic compounds within the extract or the presence of other antioxidants, leading to changes in DPPH scavenging activity. Further research is needed to fully understand the mechanisms by which temperature affects TPC and the temperature-dependent changes in DPPH inhibition and to determine the impact on the overall antioxidant profile of EL extract. This underscores the importance of considering the complex interplay of factors beyond TPC when assessing the antioxidant activity of plant extracts.

We found that the concentration of EL extract significantly influenced the duration of semen storage and the preservation of sperm motility, viability, and other important quality parameters. Notably, a concentration of 15 mg/mL exhibited the most favorable impact on semen quality, maintaining it for up to 48 h. This improvement in semen quality can be attributed to the phenolic compounds present in the EL extract that are known to stimulate mitochondrial function. This stimulation results in increased sperm motility by enhancing energy generation rates during cellular respiration processes (34). Furthermore, the antioxidant properties of the EL extract, particularly its ability to scavenge free radicals, are supported by the lower MDA levels observed in the semen supplemented with EL extract, particularly at 24 and 48 h of storage. This improvement in sperm quality is further supported by the lower MDA levels observed, suggesting that EL extract effectively scavenges free radicals and reduces oxidative stress damage during the early storage stages. However, after 72 h of storage, the antioxidant capacity of the EL extract may be depleted, as indicated by the less pronounced difference in MDA levels between the treatment groups. This suggests that while EL extract is effective in maintaining sperm quality for up to 48 h, its effectiveness may diminish over longer storage periods.

While previous studies highlight the crucial role of antioxidant enzymes like SOD, CAT, and glutathione peroxidase (GPx) in protecting semen from oxidative stress (35–37), our study did not detect significant differences in SOD and CAT activity across the different treatment groups (Table 4). This finding suggests that the primary role of SOD and CAT may be intracellular, protecting sperm cells from oxidative damage within their own environment. Previous research indicates SOD is pivotal in scavenging free radicals by converting O_2_^−^ radicals into H_2_O_2_, while the subsequent transformation of H_2_O_2_ into water molecules is facilitated by CAT and GPx (35). GPx is reported as the dominant antioxidant enzyme in chicken semen (35, 37), potentially explaining why the observed lower levels of MDA, a marker of oxidative stress, were not accompanied by significant differences in SOD and CAT activities. Similar findings have been reported in previous studies (25, 38), where glutathione supplementation in chicken semen for cooled storage did not result in statistically significant differences in GPx and SOD activities in seminal plasma, while the total antioxidant capacity showed a significant difference (*p* < 0.05). Additionally, variations in MDA levels were observed between chicken semen with high and low motility, despite no differences in SOD and CAT activity (25). These findings suggest that while the presence of efficient antioxidant systems, including SOD and CAT, is important for protecting sperm cells from oxidative stress, other, potentially more dominant, antioxidant mechanisms might be at play. This emphasizes the complexity of antioxidant defense within semen and highlights the need for further investigation into the interplay of various antioxidant pathways, including both intracellular and extracellular mechanisms, to fully understand the impact of EL extract on semen quality during storage.

Besides their antioxidant properties, the EL extract possesses bactericidal properties attributed to its active components such as quassinoids and eurycomanone. These compounds induce membrane damage that leads to increased ion permeability, substance leakage, and disruption of bacterial enzymatic systems (39). These mechanisms may also affect sperm cell membranes, explaining why excessive supplementation of EL extract (20 mg/mL) may be detrimental. A study on *Rosmarinus officinalis* essential oil supplementation effects on rooster sperm motility at 4°C revealed that the highest level of supplementation (870 μg/mL) resulted in increased sperm cell death due to cell membrane destruction, highlighting the bactericidal properties of this plant extract as well (40). Additionally, elevated antioxidant concentrations have been reported to potentially dehydrate sperm cells within the solution, leading to hypertonic conditions (41, 42). High concentrations of phenolic compounds and flavonoids can negatively affect mitochondrial cellular respiration by directly reacting with mitochondrial membranes, and this impairs ATP production and the function of complex I and coenzyme Q-binding that is necessary for NADH electron transfer (43, 44). Excessive levels of quercetin, a flavonoid, can also inhibit sperm motility and viability by decreasing or halting Ca^2+^-ATPase activity that is crucial for maintaining sperm cell motility (45).

The present study demonstrated that supplementation of semen extender with the EL extract at a concentration of 15 mg/mL significantly improved the fertility rate in native Thai rooster semen stored for 24 h. This enhancement in fertility can be attributed to the antioxidant properties of the EL root extract that positively affected sperm quality (13). Improved semen quality, particularly in terms of sperm motility and viability, likely contributed to the prolonged survival of sperm in the sperm storage tubules (SSTs) of hens and facilitated a higher rate of migration toward fertilization (46). However, the beneficial effects of EL extract supplementation on sperm fertility were not observed at a semen storage duration of 48 h, where no significant differences in fertility rates were found between the treated and control groups, even though the sperm quality in the treatment group was superior to that in the control group. There are two potential reasons for this outcome. First, while the viability of sperm after 48 h of storage in the treatment group remained high (approximately 80%), the PMOT was around 60%, which may be too low to allow successful migration to the SSTs (47). Second, the decline in antioxidant capacity after 48 h of semen storage could be insufficient to neutralize the increased levels of oxidative state associated with longer semen storage periods (10). Future research should explore strategies to extend the antioxidant efficacy of the EL extract and other potential supplements to improve semen quality over longer storage periods.

## 5 Conclusion

Supplementation of sperm cooling medium with EL extract at a concentration of 15 mg/mL enhanced semen quality during cold storage for up to 48 h, with a decrease in MDA levels in cooled semen. In addition, EL extract at a concentration of 15 mg/mL effectively enhanced fertility rates of native Thai rooster semen when stored for up to 24 h.

## Data Availability

The original contributions presented in the study are included in the article/supplementary material, further inquiries can be directed to the corresponding author.
